# Reverse Total Shoulder Arthroplasty for Proximal Humerus Nonunion

**DOI:** 10.3390/jcm14145130

**Published:** 2025-07-18

**Authors:** James Tyler Frix, Maria Kammire, Nainisha Chintalapudi, Patrick Connor

**Affiliations:** 1 Atrium Health Carolinas Medical Center, 2001 Vail Ave, Charlotte, NC 28207, USA; maria.kammire@atriumhealth.org (M.K.); nainisha.chintalapudi@atriumhealth.org (N.C.); 2 OrthoCarolina, 1915 Randoph Rd, Charlotte, NC 28207, USA

**Keywords:** RTSA, reverse total shoulder arthroplasty, shoulder arthroplasty, proximal humerus fracture, fracture, nonunion, tuberosity, tuberosity repair, greater tuberosity, lesser tuberosity

## Abstract

**Background:** Surgical neck nonunions of the proximal humerus present a complex clinical challenge, especially in elderly patients with pre-existing glenohumeral arthritis. Reverse total shoulder arthroplasty (RTSA) offers a reliable treatment option in these cases; however, resection of the tuberosities may compromise joint stability, increase the risk of postoperative dislocation and compromise postoperative function. This article describes a reproducible RTSA technique that preserves and repairs the greater and lesser tuberosities, aiming to enhance construct stability and optimize outcomes. **Methods:** We present a 74-year-old female with underlying glenohumeral arthritis who underwent RTSA for a symptomatic surgical neck nonunion via an extended deltopectoral approach. The nonunion is first mobilized, and tuberosity osteotomies are performed. After implant placement, the tuberosities are secured to the implant, to each other, and to the humeral shaft. A cerclage suture is also passed circumferentially to reinforce the repair and prevent posterior gapping. **Results:** The patient regained her pre-injury level of function by her last follow-up. She had pain-free, active forward elevation to 110 degrees and radiographic evidence of maintained tuberosity reduction and healing. There was no evidence of instability. **Conclusions:** In conclusion, incorporating tuberosity preservation and repair into RTSA for proximal humerus nonunion may reduce dislocation risk and improve functional recovery in elderly, low-demand patients.

## 1. Introduction

Patients with pre-existing glenohumeral arthritis are at an increased risk of nonunion in proximal humerus fractures as motion can lead to translation through the fracture site rather than the joint itself [1]. It is well established that surgical neck nonunions are significantly debilitating for this population, and limit functional use of the shoulder [2,3,4]. Various surgical treatments have been proposed, one of which is the utilization of reverse total shoulder arthroplasty (RTSA) which has shown promising clinical outcomes [5,6,7]. Despite these positive results, there is data to suggest that there is a higher rate of post-operative shoulder dislocation associated with intraoperative resection of the greater and lesser tuberosities during this approach [7]. The goal of this article is to describe a method for the preservation of the tuberosities and, thus, the rotator cuff attachment in order to maximize joint stability following RTSA for proximal humerus nonunion.

## 2. Surgical Technique

This technique describes the creation of greater and lesser tuberosity osteotomies for improved exposure, the implantation of both the glenoid and humeral implants, and a robust tuberosity reconstruction which includes cerclage fixation to increase stability and retain rotator cuff function. The senior author’s preferred method for positioning is beach-chair with a sterile arm holder. A standard deltopectoral approach is performed. Important aspects of this exposure include taking down the leading 1 cm of the pectoralis major tendon and clearing subdeltoid adhesions to allow maximal exposure. The axillary nerve is identified medially anterior to the subscapularis and medial to the conjoined tendon as well as its extension posteriorly and laterally in the subdeltoid plane. The rotator interval is entered, and a Darrah retractor is placed in the interval to assist with visualization of the tuberosities.

**Figure 1:**
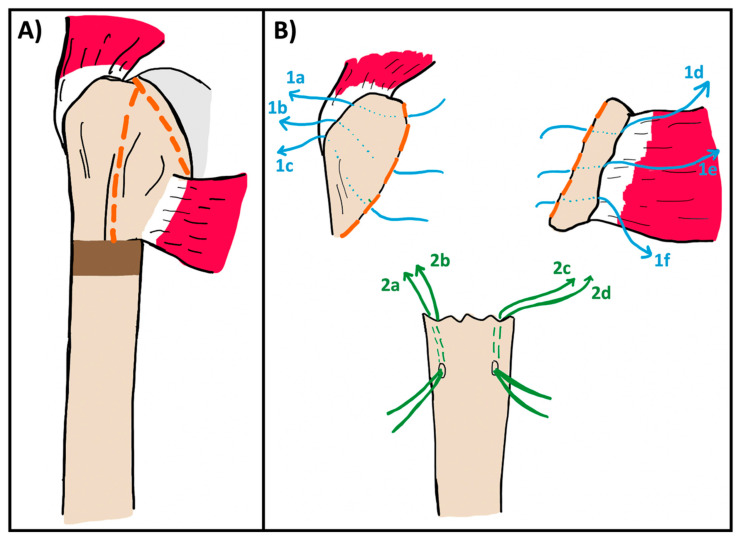
(**A**) Illustration of a proximal humerus nonunion showing the planned greater and lesser tuberosity osteotomy through the bicipital groove and humeral head osteotomy (orange dashed lines). (**B**) Suture configuration through the greater (1a–c, blue) and lesser (1d–f, blue) tuberosity segments. Three #5 ethibond sutures are placed using an “inside-out” technique through each osteotomized segment and out the bone–tendon junction of each tuberosity. Two tunnels are then drilled in the humeral shaft and two sutures are passed through each (2a–d, green) for later repair.

The greater/lesser tuberosity osteotomy begins at the bicipital groove anteriorly (Figure 1A). The lesser tuberosity osteotomy is completed first using a large osteotome. After the osteotome penetrates the anterior cortex at the bicipital groove, it is angled medially and inferiorly to separate the lesser tuberosity from the humeral head. Completing the lesser tuberosity osteotomy allows for mobilization and debridement of the surgical neck nonunion, taking care to preserve any salvageable subscapularis tendon for repair. This tissue is then tagged along the bone–tendon juncture using three #5 Ethibond sutures (Ethicon Inc., Raritan, NJ, USA) (or any non-absorbable suture of the surgeon’s choice) which are clamped for later repair. This suture is passed, using an inside out technique, through the osteotomized lesser and then out through the subscapularis tendon (Figure 1B). The subscapularis is further mobilized with the use of heavy scissors through the capsule and superior glenohumeral ligament on the upper border of the subscapularis and the anteroinferior edge of the glenoid. A finger is placed over the axillary nerve for protection during inferior dissection. In fracture work, the nerve may be more scarred down than in standard arthroplasty cases, so careful dissection is warranted.

Once the lesser tuberosity has been freed and tagged, and the subscapularis has been adequately mobilized, attention is turned to the greater tuberosity. A large osteotome is again used following a similar path through the bicipital groove and then angling laterally and superiorly to detach the greater tuberosity, preserving the supraspinatus and infraspinatus attachments. Care must be taken during this step to avoid skiving with the osteotome and cutting into the glenoid. Once the greater is freed, it is also tagged with three #5 Ethibonds using an inside-out technique at the bone–tendon junction for later repair (Figure 1B). The remaining fracture fragments and humeral head are removed. In some cases, the humeral head may remain attached to the calcar medially and posteriorly, and an osteotomy at the anatomic neck can be used to release it (Figure 1A). Two drill tunnels are then placed in the humeral shaft just distal to the nonunion site and two #5 Ethibond sutures are passed through each drill tunnel using an outside in technique. These sutures are then passed through the bone–tendon junction of the greater and lesser tuberosities and tagged for later repair (Figure 1B).

**Figure 2:**
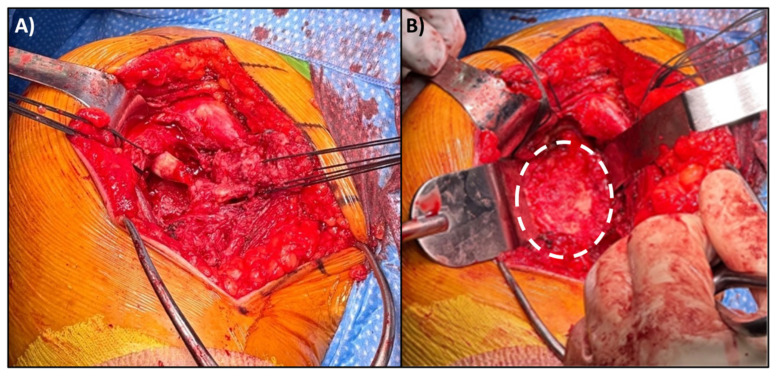
(**A**) Greater and lesser tuberosity osteotomies in our patient, tagged with #5 Ethibond sutures. For orientation, the greater tuberosity is lateral (left of image) and the lesser tuberosity is medial (right of image). (**B**) Glenoid exposure for glenosphere implantation; the glenoid has been outlined with a white, dashed oval. Typically, the greater and lesser tuberosity osteotomies allow for ample exposure of the glenoid, and the tagging sutures can be used to assist with soft tissue retraction.

Osteotomies of the tuberosities and removal of the humeral head provides ample glenoid exposure for routine glenosphere insertion using the surgeons’ preferred choice of implant (Figure 2). Once this is placed, attention is turned towards humeral component and polyethylene trialing. A trauma-specific humeral stem—the Lima SMR Trauma in this case—is used as this allows for multiple sites of suture fixation through the stem. Trials of increasing polyethylene size are placed until the shoulder is no longer easily reducible, then one size is subtracted for the final component. This method ensures stability while preventing overstuffing. The trial construct should be stable to manual stress and shuck testing prior to proceeding final implant placement.

**Figure 3:**
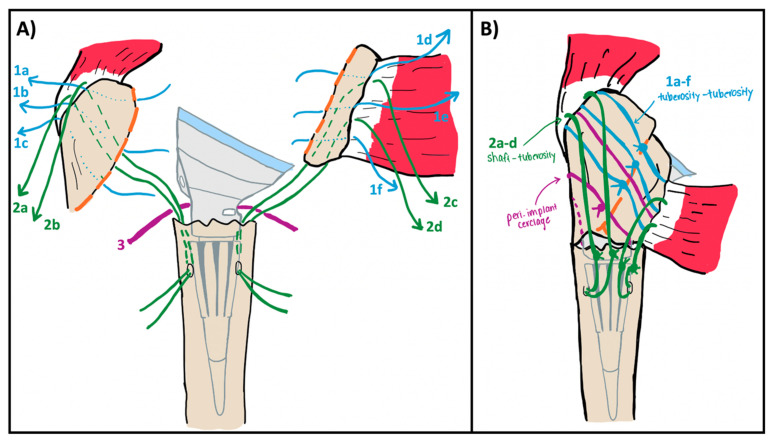
(**A**) Illustration of final humeral implant placement with a “passing” suture (3, purple) being seated in the implant medial groove and around the posterior aspect of stem. This passing suture will be used later in the case to pass an Arthrex Fibertape cerclage. (**B**) Illustration of tuberosity fixation using the cerclage, tuberosity sutures (1a–f, blue), and humeral shaft sutures (2a–d, green). Of note, the tails of the tuberosity sutures (1a–f) should be routed anteriorly before tightening the cerclage so that they can be tied to themselves. The orange dashed line corresponds with the osteotomy sites.

As the final humeral implant is introduced into the canal, a single Ethibond suture is passed posteriorly around the humeral stem as a passing suture for subsequent cerclage (Figure 3A). This is performed prior to final spacer and polyethylene placement to ensure proper placement within the implant groove. Once the implant is seated, the lesser and greater tuberosity osteotomies are meticulously reapproximated using the cerclage and the previously passed tuberosity and humeral shaft sutures (Figure 3B).

**Figure 4:**
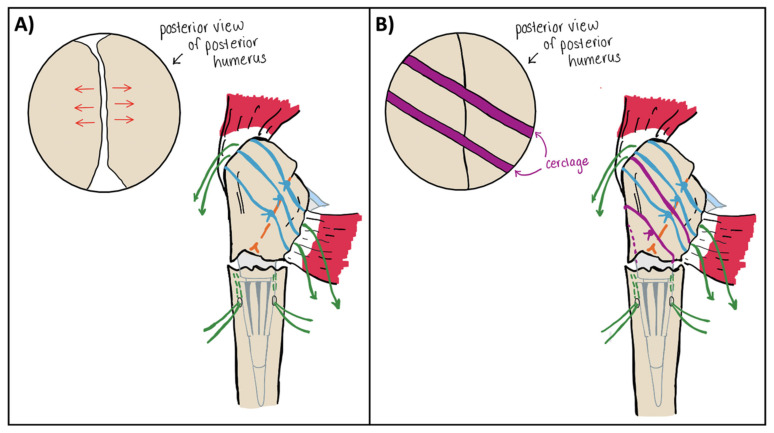
(**A**) Illustration of tuberosity approximation (orange dashed line) with the tuberosity sutures (blue sutures). Note that without a cerclage (purple suture) you can have posterior gapping (inset, red arrows) due to anterior over tensioning of these sutures. (**B**) Following cerclage placement, tuberosity sutures are tied in the same fashion; however, posterior gapping is prevented (inset). Note, green sutures are the humeral shaft sutures that will be tied down last.

**Figure 5:**
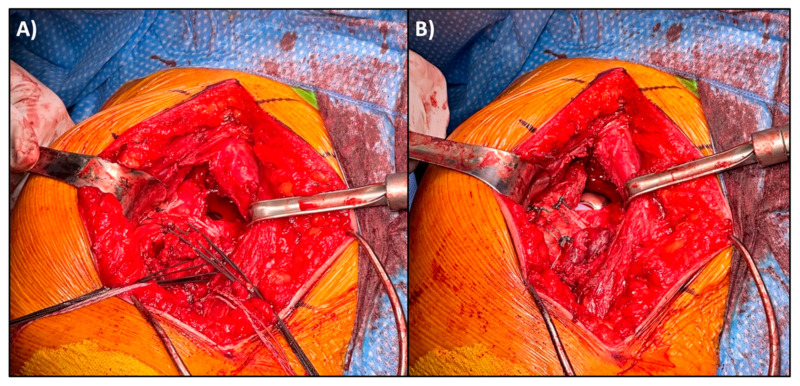
(**A**) Reapproximation of the greater and lesser tuberosity osteotomies following final implant and cerclage placement and final tensioning of the Fibertape cerclage (white suture) to fixate the tuberosities. (**B**) Following Fibertape cerclage, the 3 tagging sutures from each tuberosity are tightened and tied down anteriorly. The white suture is the cerclage suture tape that has been passed around the humerus just distal to the humeral head osteotomy for additional stability.

The cerclage suture—in this case Arthrex FiberTape (Arthrex, Naples, FL, USA)—is passed using the previously placed Ethibond suture. Care is taken to ensure the cerclage sits in the implant groove at the medial neck. If desired, a second passing suture can be pulled around the tuberosities with the cerclage to allow a second cerclage pass. The previously placed Ethibond sutures at the bone–tendon interface of the greater and lesser tuberosities are then passed through anchor holes in the humeral implant stem. Of note, there is typically only room for 4 of these 6 sutures to pass through the stem. At this point the cerclage is tensioned to fixate the greater and lesser tuberosities to each other. It is critical to pass sutures 1a–f through the implant and bring the tails out anteriorly before the cerclage is tightened, as tightening of the cerclage will lead to loss of access to the stem. After cerclage tensioning, the six tuberosity sutures (1a–f) are tied to their tails anteriorly, tightly securing the tuberosities to the implant. As these sutures are brought together, the previously tightened cerclage serves to prevent posterior gaping of the tuberosities (Figure 4). At this point, the tuberosities are fixed to each other and to the implant. Finally, the sutures passed through the humeral shaft are tied, fixing the construct to the humeral shaft. With this technique, the tuberosities are secured to one another, the implant, and the humeral shaft to achieve maximal stability (Figure 5). Additional Ethibond sutures are placed in a figure-of-eight fashion as needed for further reinforcement of the subscapularis tendon. At this point, the final construct should move as a singular unit with forward flexion and internal/external rotation. The wound is then thoroughly irrigated and closed in a layered fashion.

## 3. Results

**Figure 6:**
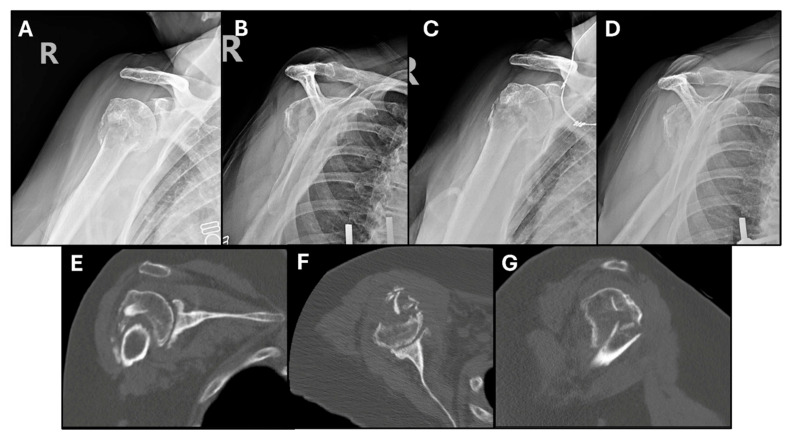
Selected radiographs and CT scan cuts from our patient’s right (R) shoulder. (**A**,**B**) Initial injury radiographs of the right shoulder demonstrating surgical neck fracture. (**C**,**D**) Radiographs obtained after 6 weeks of nonoperative management, showing interval varus displacement and minimal callus formation. (**E**–**G**) Coronal, axial and sagittal CT cuts at 10 weeks post-injury depicting no significant bridging callus at the fracture site. Underlying glenohumeral arthritis is also observed.

We present the case of a 74-year-old, right-hand dominant, Caucasian female who sustained a displaced surgical neck fracture of the right proximal humerus (Figure 6A,B). Prior to this injury, she functioned independently and was able to perform all daily activities of living but she did use a rolling walker for gait assistance. Her medical co-morbidities included obesity (BMI 35), hypertension, hyperlipidemia, hypothyroidism, osteopenia, stage 2 chronic kidney disease, and obstructive lung disease, and multiple spine surgeries for lumbar pathology. She initially was treated at an outside facility and underwent a trial of non-operative management in a cuff and collar for 6 weeks followed by physical therapy; however, after 11 weeks of ongoing conservative measures there was continued lack of radiographic healing and progressive varus displacement (Figure 6C,D). A CT scan was subsequently obtained, and this showed no significant bridging callus at the fracture site and underlying glenohumeral arthritis (Figure 6E–G) and she was referred to our office for discussion of surgical management. At this point, the patient was three months out from her initial injury with significant ongoing pain and functional limitations. She was having issues supporting herself with her walker and performing overhead activities such as doing her hair. She was only able to achieve 70 degrees of active forward elevation, 10 degrees of external rotation, and internal rotation to the lumbosacral junction. She was indicated for a right RTSA and underwent the procedure described above. An STD glenoid baseplate and a 40 mm glenosphere were utilized and offset inferiorly. A Lima finned, uncemented diaphyseal-fit stem sized to 15 mm was placed in addition to one +9 spacer and a +6 polyethylene to idealize periarticular soft-tissue tension.

**Figure 7:**
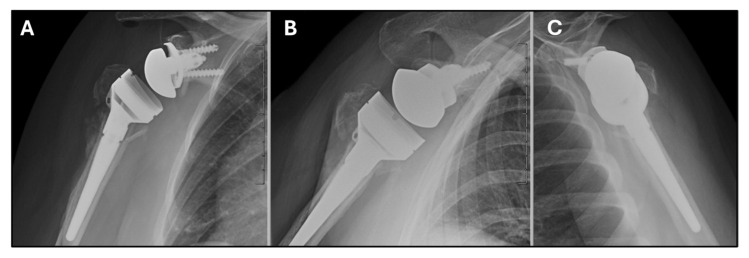
Radiographs of the right shoulder at 6 weeks post-op showing reverse total shoulder arthroplasty with maintained tuberosity reduction and alignment. (**A**) Grashey view. (**B**) Velpeau view. (**C**) Scapular Y view.

Overall, she had an uncomplicated post-operative course and reported gradual resolution of pain. She was seen back in the office at 2 weeks for suture removal. At the 6-week follow-up, her radiographs showed evidence of maintained tuberosity reduction and alignment (Figure 7), and physical therapy was started at this time. At 3 months she had improvement in her pain but continued to have some weakness with forward elevation. By her final in-office visit at 5 months postop, she was able to achieve 110 degrees of active forward elevation, unfortunately we do not have internation and external rotation values recorded in the chart for this final visit. At this visit she reported return to her pre-injury activities including independence with ADLs, ambulation with a rolling walker for assistance, and the ability to perform overhead hygiene such as curling her hair.

## 4. Discussion/Conclusions

Humeral surgical neck nonunion in elderly, low-demand patients can be successfully treated with reverse total shoulder arthroplasty. Repairing the greater and lesser tuberosities can improve outcomes by preserving rotator cuff strength and may provide increased construct stability when compared to resection alone and, thus, lessen the risk of future shoulder dislocation [7]. The above technique outlines a reproducible approach to exposure and reconstruction in patients with this pathology and has proven to be quite reliable in the senior surgeon’s own practice. Of note, variations of this technical tip for suture reconstruction of the tuberosities can be applied to reinforce the repair of acute proximal humerus fractures with tuberosity involvement, at the surgeon’s discretion, in hopes of preventing superior tuberosity escape. In these cases, the sutures can be passed through the plate (instead of the stem) to once again provide security to each other, the implant, and the distal humeral shaft.

## Data Availability

The original contributions presented in this study are included in the article. Further inquiries can be directed to the corresponding author.

## Abbreviations

The following abbreviations are used in this manuscript:

RTSA	Reverse total shoulder arthroplasty
